# A Preliminary Randomized Trial on the Efficiency and Clinical Value of a Cementless Screw-Retained Implant Workflow in Single-Implant Restorations

**DOI:** 10.3390/jfb16100378

**Published:** 2025-10-10

**Authors:** Sang-Yoon Park, Sung-Woon On, Tae-Yoon Park, Seoung-Won Cho, Sang-Min Yi, Soo-Hwan Byun, Hyun-Sook Han, Lee-Kyoung Kim, Byoung-Eun Yang

**Affiliations:** 1 Department of Oral and Maxillofacial Surgery, Hallym University Sacred Heart Hospital, Anyang 14068, Republic of Korea; psypjy0112@naver.com (S.-Y.P.); taeyoon313@gmail.com (T.-Y.P.); queen21c@gmail.com (S.-M.Y.); purheit@daum.net (S.-H.B.); 2 Department of Artificial Intelligence and Robotics in Dentistry, Graduate School of Clinical Dentistry, Hallym University, Chuncheon 24252, Republic of Korea; drummer0908@hanmail.net (S.-W.O.); kotneicho@gmail.com (S.-W.C.); hshan@hallym.or.kr (H.-S.H.); 2gyoung2@naver.com (L.-K.K.); 3 Institute of Clinical Dentistry, Hallym University, Chuncheon 24252, Republic of Korea; 4 Dental Artificial Intelligence and Robotics R&D Center, Hallym University Medical Center, Anyang 14066, Republic of Korea; 5 Division of Oral and Maxillofacial Surgery, Hallym University Dongtan Sacred Heart Hospital, Hwaseong 18450, Republic of Korea; 6 Department of Prosthodontics, Hallym University Sacred Heart Hospital, Anyang 14068, Republic of Korea

**Keywords:** digital dentistry, combined screw- and cement-retained prosthesis (CSCRP), cementless screw-retained prosthesis (CL-SRP), intraoral scanning, chairside efficiency, peri-implant soft tissue, marginal bone loss, implant prosthodontics

## Abstract

This randomized controlled clinical trial compared a conventional combined screw- and cement-retained prosthesis (CSCRP) workflow (control group) with a fully digital cementless screw-retained prosthesis (CL-SRP) system (test group) for single posterior implant restorations. A total of 40 implants in 35 patients were allocated to either workflow. Clinical procedure times, prosthetic accuracy, peri-implant soft tissue changes, and marginal bone loss (MBL) were assessed. The test group demonstrated significantly shorter total prosthetic time (*p* < 0.001) and impression-taking time (*p* < 0.001) compared with the control group. Prosthetic adjustment time (*p* = 0.211) and adjustment volume (*p* = 0.474) did not differ significantly. Gingival shape changes were likewise not statistically significant (*p* = 0.966). MBL was significantly lower in the test group (*p* < 0.05). From a prosthetic standpoint, both workflows yielded clinically acceptable outcomes; however, the digital CL-SRP approach improved procedural efficiency and early peri-implant bone preservation without compromising prosthetic quality. This trial had inherent limitations, including a short follow-up duration, a relatively small sample size, combined test conditions, and restriction to single posterior implants. Therefore, further long-term studies are warranted to confirm durability and broader clinical applicability.

## 1. Introduction

Dental implants are widely regarded as a well-established and predictable treatment modality for tooth replacement [1]. Within implant prosthodontics, the choice of prosthetic retention type—cement-retained or screw-retained—has long been a critical determinant of clinical success and long-term maintenance. Cement-retained prostheses offer esthetics and a passive fit, but pose biological risks associated with residual cement. In contrast, screw-retained prostheses ensure retrievability but may lead to mechanical complications, such as screw loosening or fracture [2,3]. To overcome these limitations, the combined screw- and cement-retained prosthesis (CSCRP) was introduced, aiming to integrate the advantages of both approaches [4,5].

In recent years, rapid advances in digital dentistry—including intraoral scanning, computer-aided design/computer-aided manufacturing (CAD/CAM), and fully digital workflows—have revolutionized implant prosthodontics. These technologies enhance precision, reduce the number of clinical visits, and improve patient comfort by eliminating the need for conventional impressions and casts [6,7,8,9]. Digital workflows have consistently demonstrated both accuracy and efficiency, which has contributed to their increasing adoption in daily prosthodontic practice.

One notable development reflecting this trend is the cementless screw-retained prosthesis (CL-SRP), a system that integrates digital technology with cementless prosthetic design in implant dentistry. Unlike conventional screw-retained restorations, which are directly attached to the abutment, the CL-SRP incorporates a titanium base and a CAD/CAM-fabricated internal connector. This two-component system allows occlusal screw access to the final restoration, eliminating cement dependence while ensuring retrievability [10,11]. The CL-SRP design may offer potential advantages such as improved seating accuracy, reduced micromotion, enhanced stability, and biological safety by altogether avoiding cement-related complications.

Although CL-SRP use has become increasingly common in clinical practice—particularly for posterior single-tooth implants, where treatment efficiency and retrievability are essential—robust comparative evidence regarding its long-term effectiveness remains limited. The lack of randomized controlled trials specifically evaluating CL-SRP performance relative to established CSCRP protocols represents a significant gap in the current clinical evidence. Previous studies have primarily investigated digital versus conventional impressions [12,13,14] or screw versus cement retention [15,16] independently; however, few have assessed the combined impact of digital workflow integration and cementless prosthetic design in a clinical setting.

Accordingly, this randomized controlled clinical trial was conducted to compare a fully digital CL-SRP workflow with the conventional CSCRP protocol for posterior single-tooth implant restorations. The study focused on key clinical outcomes, including procedural efficiency (impression-taking and prosthesis placement time), prosthetic accuracy, peri-implant soft tissue response, and marginal bone changes. The null hypothesis was that there would be no significant differences between the CL-SRP and CSCRP workflows with respect to these outcomes. The alternative hypothesis was that the CL-SRP workflow would demonstrate superior procedural efficiency while maintaining comparable biological outcomes.

## 2. Materials and Methods

### 2.1. Study Design

This randomized controlled clinical trial was designed to evaluate the clinical efficiency and outcomes of a conventional CSCRP workflow and a fully digital CL-SRP workflow in posterior single-implant restorations. A total of 40 implants in 35 patients were included and randomly allocated to two groups: 20 in the control group (CSCRP, conventional workflow) and 20 in the test group (CL-SRP, digital workflow).

The sample size was calculated using G*Power software (version 3.1.9.7, Heinrich-Heine-Universität Düsseldorf, Germany) based on pilot in vitro data. Assuming a significance level of 0.05, an effect size of 1.2, a statistical power of 0.95, and a 1:1 allocation ratio, a minimum of 32 implants (16 per group) was required. To compensate for a potential 20% dropout rate, the final target sample size was set at 40 implants (20 per group) [17].

The randomization sequence was computer-generated with a 1:1 allocation. An independent, unblinded coordinator managed the allocation list and performed the randomization. Personnel responsible for enrollment and intervention assignment were not involved in sequence generation. Participants and outcome assessors (data analysts) were blinded to group allocation to minimize performance bias, whereas clinician blinding was not feasible due to inherent procedural differences.

All implant surgeries were performed by oral and maxillofacial surgeons using a one-stage surgical protocol, followed by a healing period of approximately 12 weeks. Two prosthodontists completed the final prosthetic procedures. In both groups, implants were placed using the Osstem TS III system (Osstem Implant Co., Ltd., Seoul, Republic of Korea), with diameters ranging from 4.0 to 5.0 mm and lengths of 8.5 to 11.5 mm. All implants were positioned approximately 1 mm subcrestally in accordance with the manufacturer’s guidelines to ensure consistency for subsequent marginal bone level evaluation. Prostheses in the test group were fabricated using the Digital Retained screw system (DR screw system, Osstem Implant Co., Ltd., Seoul, Republic of Korea). The overall clinical observation period was approximately 6 months.

**Figure 1:**
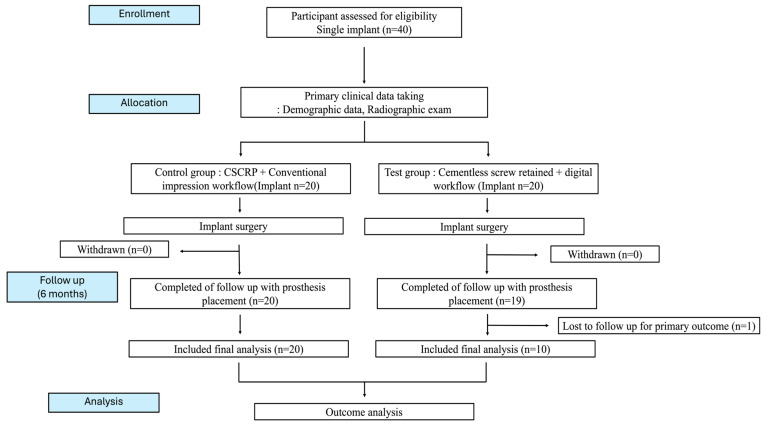
CONSORT diagram.

The study was conducted in compliance with the Consolidated Standards of Reporting Trials (CONSORT) guidelines to ensure methodological rigor and transparency. Ethical approval was obtained from the Institutional Review Board of Hallym University (IRB No.: HALLYM IRB 202405001001). The trial was registered with the Korea National Clinical Trial (KNCT) registry (KCT0009159). All participants provided written informed consent prior to enrollment. Participant confidentiality was strictly maintained, and all personal identifiers were removed (Figure 1).

### 2.2. Patient Selection

Patients were eligible if they were ≥19 years of age with completed jaw growth and required single-tooth implant placement due to partial edentulism. Only patients who provided written informed consent were enrolled. Eligible participants were non-smokers or light smokers (≤20 cigarettes/day) with no systemic or local contraindications to implant surgery. Exclusion criteria included pregnancy, uncontrolled systemic diseases (e.g., uncontrolled diabetes, cardiovascular disease), high-risk oral lesions, or mucosal pathology at the intended implant site. Patients with a history of long-term medication use affecting bone metabolism (e.g., bisphosphonates or corticosteroids), prior head and neck radiotherapy, or ongoing chemotherapy were excluded. Additional exclusion criteria included heavy smoking (>20 cigarettes/day), poor oral hygiene, untreated periodontal disease or caries, and any other medical or dental condition deemed unsuitable by the investigators.

### 2.3. Clinical and Prosthetic Procedures by Group

#### 2.3.1. Control Group (CSCRP System with Conventional Workflow)

**Figure 2:**
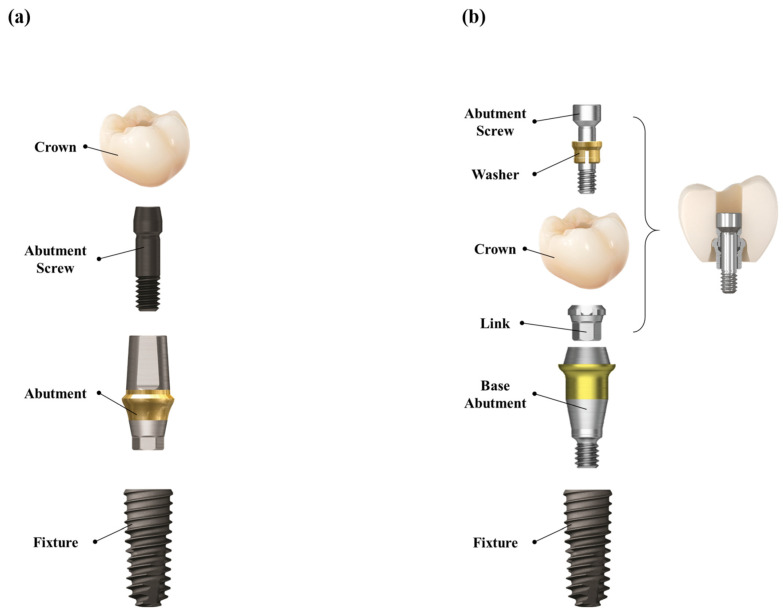
Schematic diagram of the two dental implant systems used in our study. (**a**) CSCRP system (control group). (**b**) CL-SRP system (test group).

**Figure 3:**
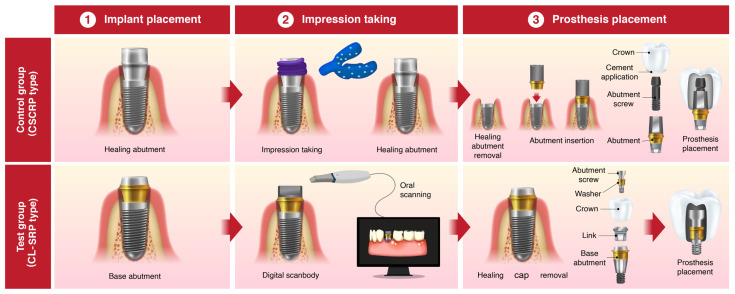
Detailed overview of the clinical procedures and workflow differences between the CSCRP and CL-SRP system groups.

In the control group, the healing abutment was removed, and a polyvinyl siloxane (PVS) impression was made using a stock tray and impression coping (Delikit Regular Body and Delikit Light Body, Sherpa Korea, Republic of Korea). A custom titanium abutment was fabricated based on the definitive cast. A monolithic zirconia crown was then cemented to the abutment using a self-adhesive resin cement (RelyX U200, 3M ESPE, St. Paul, MN, USA). At the time of prosthesis delivery, the abutment–crown complex was removed, thoroughly cleaned, and reinserted. The abutment–crown assembly was then torqued to 30 Ncm onto the implant fixture. The screw access channel was sealed with a flowable resin composite (Filtek Supreme Flowable Restorative, 3M, ESPE, St. Paul, MN, USA) to achieve occlusal sealing. This workflow ensured retrievability, minimized the risk of cement remnants, and provided a passive fit. (Figure 2a and Figure 3)

#### 2.3.2. Test Group (CL-SRP System with Digital Workflow)

In the test group, a prefabricated base abutment (DR screw system, Osstem Implant Co., Ltd., Seoul, Republic of Korea) was torqued to 30 Ncm and kept in position throughout the procedure. A scan body was attached, and an intraoral scanner (TRIOS 3, 3Shape, Copenhagen, Denmark) was used to obtain the digital impression. The resulting stereolithography (STL) file was transferred to the dental laboratory, where a monolithic zirconia crown was designed and CAD/CAM milled to fit precisely onto the base abutment, eliminating the need for cement. At the prosthesis delivery visit, the crown was seated and secured with a prosthetic screw torqued to 20 Ncm through the occlusal access channel. The screw access opening was sealed with a resin composite. No cement was used at any stage of the procedure, thereby eliminating the risk of cement-associated peri-implant complications. (Figure 2b and Figure 3)

### 2.4. Outcome Measures

#### 2.4.1. Clinical Procedure Times

The clinical workflow was analyzed by dividing chairside procedures into two primary intervals: impression-taking (IT) time and prosthesis placement (PP) time. IT time was defined as the interval from removal of the healing abutment to completion of the impression procedure and replacement of the healing abutment or scan body. PP time was defined as the interval from initiation of the definitive prosthesis trial to final screw tightening and sealing.

In the control group, IT time included healing abutment removal, placement of an impression coping, conventional impression using PVS, and reattachment of the healing abutment. PP time consisted of healing abutment removal, trial fitting of the abutment–crown complex, cementation, removal for excess cement cleaning, reseating with torque application at 30 Ncm, and sealing of the screw access channel.

In the test group, IT time consisted of removing the scan healing cap, verifying base abutment torque, placing the scan body, performing intraoral digital scanning, and reattaching the healing cap. PP time included healing cap removal, trial fitting of the definitive zirconia crown, screw tightening to 20 Ncm, and sealing of the screw access opening.

Prosthesis adjustment (PA) time was measured as the duration required for occlusal and proximal contact refinements during the prosthesis delivery visit. This step was consistently monitored to capture the extent of clinical adjustments required in each workflow.

Total procedural time was calculated as the sum of the PP and PA intervals, providing an overall indicator of chairside efficiency. Laboratory processing time was deliberately excluded, as the focus was on clinical procedures performed at the prosthesis delivery visit. This parameter allowed for direct comparison of the practical feasibility and potential time-saving effects of the digital protocol relative to the conventional workflow.

All time measurements were recorded by an independent assistant using a stopwatch to minimize measurement bias. The operational definitions and checkpoints for each group are presented in Table 1 to clarify the measurement protocol and facilitate reproducibility.

#### 2.4.2. Prosthetic Accuracy

Prosthetic accuracy was evaluated by quantifying the volume of zirconia crown material removed during clinical adjustment. Each crown was scanned before any adjustment and again after the adjustment procedure, with the crown consistently stabilized in the same orientation for both scans. The stereolithography (STL) files obtained before and after adjustment were superimposed, aligned, and analyzed using Geomagic Control X software (ver. 2022.0.1, 3D Systems Inc., Rock Hill, SC, USA). The software automatically calculated the volume of material removed during adjustment.

**Figure 4:**
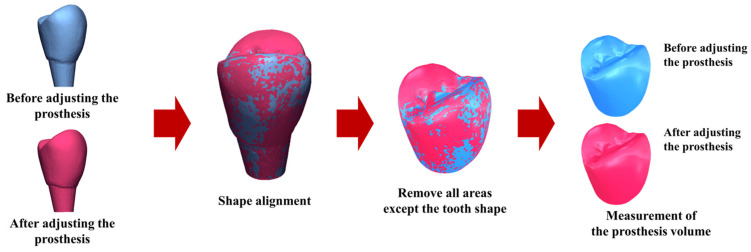
The process for evaluating the prosthesis accuracy based on the removal volume before and after adjustment of the control and test group system prostheses.

Smaller adjustment volumes indicated a superior initial fit and, consequently, greater prosthetic accuracy. This assessment was performed for both the control and test groups, allowing direct comparison of the clinical precision achieved by the conventional CSCRP workflow and the digital CL-SRP workflow. The evaluation workflow is illustrated in Figure 4.

#### 2.4.3. Peri-Implant Soft Tissue Changes

Changes in the gingival contour surrounding the implant were evaluated from the time of impression taking to the final prosthesis placement. For each patient, an intraoral scan obtained at the impression stage served as the baseline reference, and a second intraoral scan was performed at the time of definitive prosthesis placement. The two datasets were digitally trimmed to remove teeth and prosthetic components, thereby isolating the peri-implant soft tissue region of interest.

**Figure 5:**
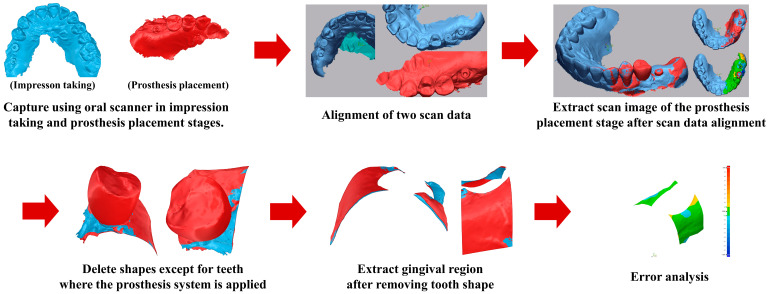
The process for evaluation comparing the gingival shape changes in the control and test group prostheses.

The trimmed surface models were superimposed using three-dimensional analysis software, and volumetric and linear changes in the gingival contour were calculated. This approach enabled the quantification of peri-implant soft tissue changes between impression-taking and final prosthesis delivery [13]. The analytical workflow is illustrated in Figure 5.

#### 2.4.4. Marginal Bone Loss (MBL) Changes

**Figure 6:**
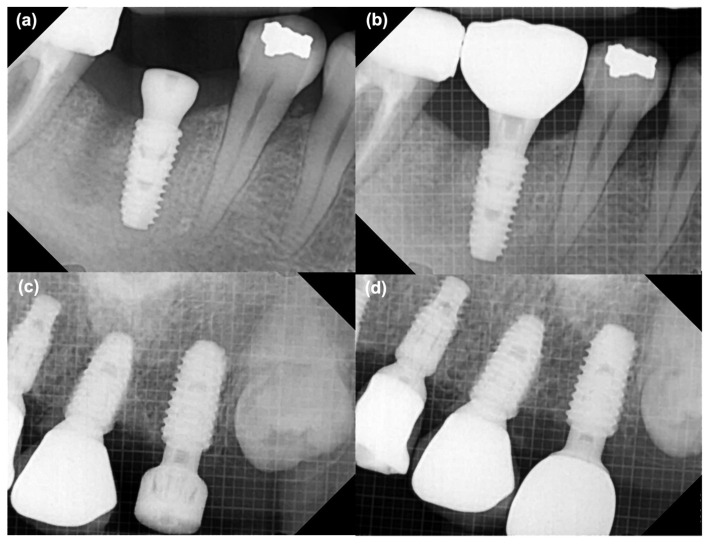
Periapical radiographs showing measurement of marginal bone levels at the time of implant impression and at the time of prosthetic placement. (**a**) Control group at baseline, (**b**) Control group at prosthesis placement time, (**c**) Test group at baseline, (**d**) Test group at prosthesis placement.

MBL around the implants was assessed using standardized periapical radiographs. Baseline bone levels were recorded immediately after implant surgery and reassessed at the time of definitive prosthesis placement. Radiographic evaluation was performed as the vertical distance from the implant shoulder to the crestal bone level on both the mesial and distal aspects of each implant. Changes in bone height between the impression stage and prosthesis delivery were calculated to quantify marginal bone remodeling. Representative examples of the measurement process are shown in Figure 6.

### 2.5. Statistical Analysis

All statistical analyses were performed with SPSS (version 25.0; IBM Corp., Armonk, NY, USA). The significance level was set at α = 0.05. Continuous variables were assessed for normality using the Shapiro–Wilk test and for homogeneity of variance. When the assumptions of normality and equal variance were met, independent *t*-tests were used to compare outcomes between the two groups. When assumptions were not met, the Mann–Whitney U test was used as a non-parametric alternative.

Only participants with complete outcome data were included in the final analysis. Missing values were not imputed; cases with incomplete data were excluded from the relevant analyses. To assess intra-examiner reliability, 20 randomly selected cases were re-measured, and intraclass correlation coefficients (ICC) were calculated.

## 3. Results

The baseline characteristics of the 40 single-implant restorations are shown in Table 2. No significant differences were observed between the control and test groups in terms of demographic variables, implant site distribution, or implant position, indicating that the groups were comparable at baseline. All implant procedures were completed successfully, and no intraoperative or postoperative complications were recorded in either group during the study period.

### 3.1. Clinical Procedure Times

**Figure 7:**
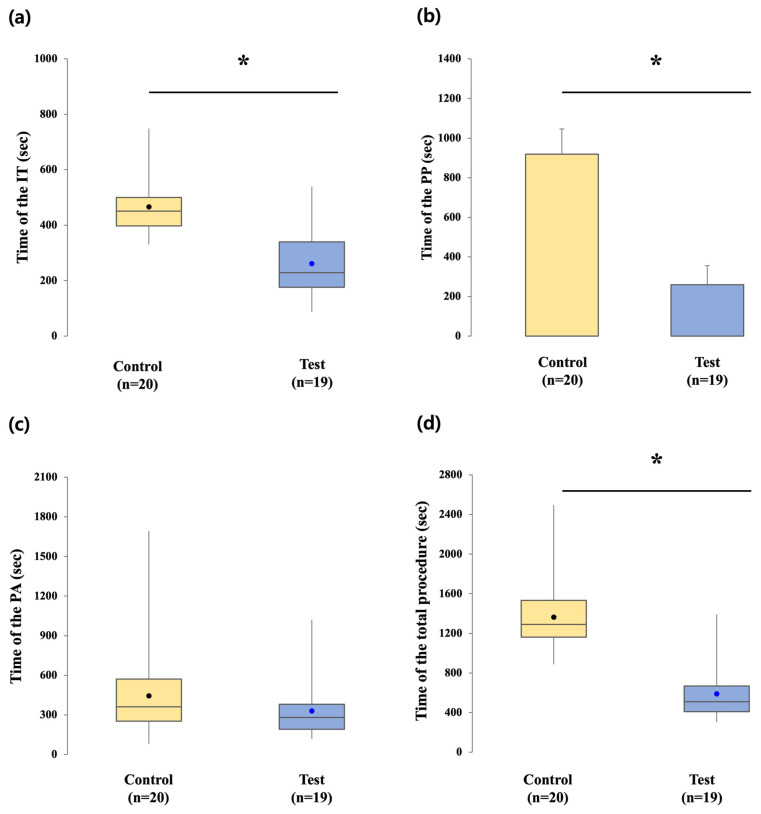
Comparison of clinical procedure times in the control and test groups. (**a**) IT time, (**b**) PP time, (**c**) PA time, (**d**) The total procedural time (PP + PA). * indicates *p* < 0.05, signifying statistical significance.

Chairside times were generally shorter in the test group than in the control group, with significant differences observed in most stages. As shown in Table 3 and Figure 7, the mean IT time was 466.10 ± 96.11 s in the control group, compared with 261.58 ± 119.11 s in the test group, indicating a significant reduction with the digital workflow (*p* < 0.001).

The mean PP time was 918.25 ± 128.46 s in the control group and 259.21 ± 95.73 s in the test group. This difference was significant, with the digital group requiring markedly less time for prosthesis placement (*p* < 0.001).

The mean PA time was 444.90 ± 352.37 s in the control group and 329.58 ± 228.34 s in the test group. Although the digital group required less adjustment on average, the difference was not significant (*p* = 0.211).

When PP and PA times were combined, the mean total prosthetic time was 1363.15 ± 347.11 s in the control group compared with 588.79 ± 270.32 s in the test group. This difference was statistically significant (*p* < 0.001).

### 3.2. Prosthetic Accuracy

**Figure 8:**
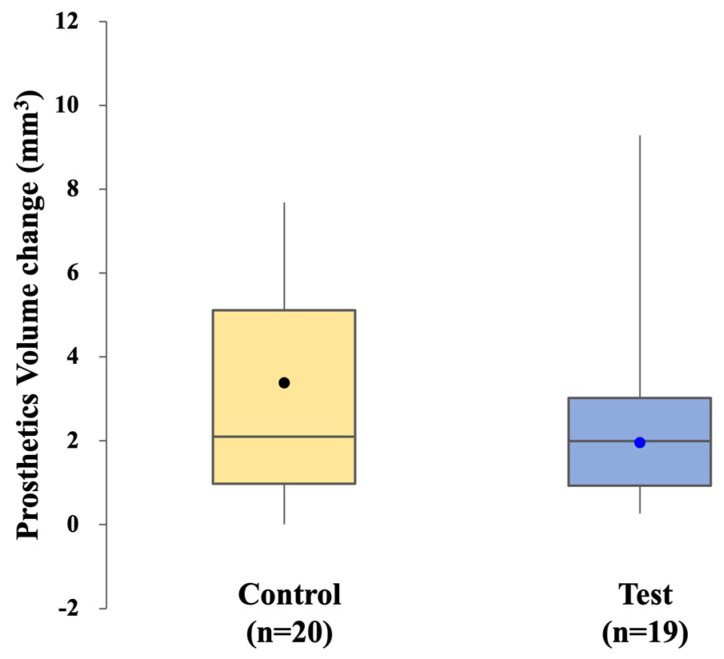
Comparison of prosthetic volume changes in the control and test groups.

The mean adjustment volume was 3.38 ± 2.46 mm^3^ in the control group and 1.96 ± 1.27 mm^3^ in the test group. The difference was not significant (*p* = 0.474) (Figure 8).

### 3.3. Peri-Implant Gingival Shape Changes

**Figure 9:**
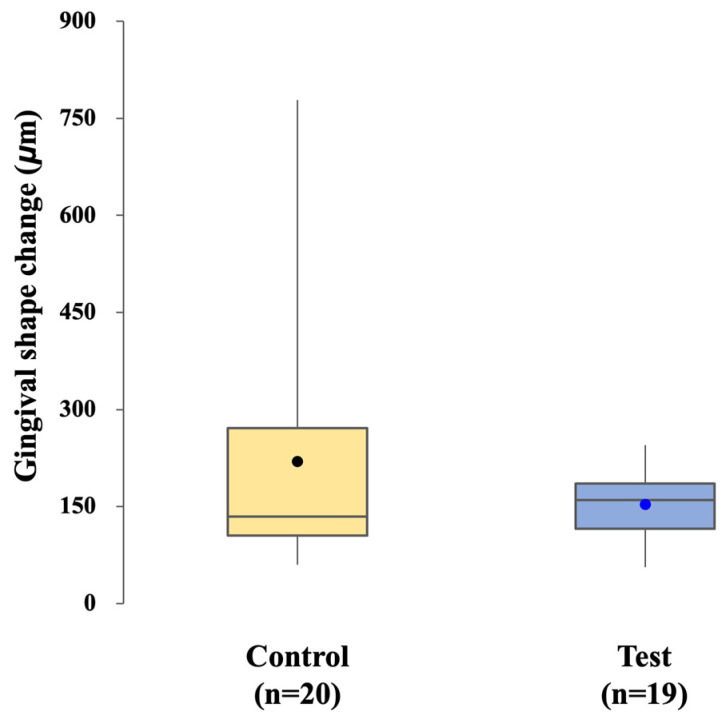
Comparison of gingival shape changes in the control and test groups.

The mean gingival contour change was 219.33 ± 187.79 µm in the control group and 153.45 ± 50.16 µm in the test group. The difference was not significant (*p* = 0.966) (Figure 9).

### 3.4. MBL

**Figure 10:**
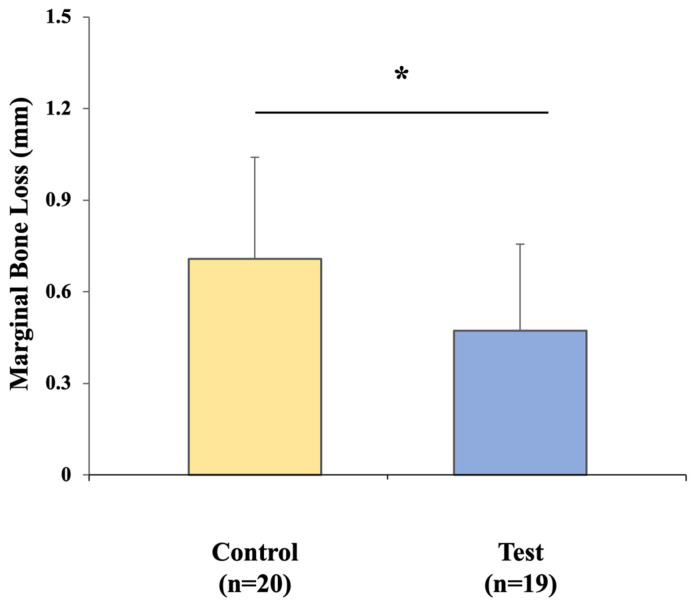
Comparison of MBL in the control and test groups. * indicates *p* < 0.05, signifying statistical significance.

Marginal bone loss averaged 0.71 ± 0.33 mm in the control group and 0.47 ± 0.28 mm in the test group, with a significant difference favoring the digital workflow (*p* < 0.05) (Figure 10). Intra-examiner reliability for MBL measurements demonstrated an ICC of 0.88.

## 4. Discussion

This randomized clinical trial demonstrated that prosthetic accuracy—measured by the extent of clinical adjustment required for the definitive crown—was comparable between the conventional CSCRP workflow and the fully digital CL-SRP workflow. Both groups required similar adjustments, and no statistically significant difference was observed in either the adjustment time or the volume of material removed. These findings suggest that the digital workflow can accurately reproduce implant positions and fabricate restorations with precision comparable to conventional methods. Previous studies have also demonstrated that intraoral scanning, combined with CAD/CAM fabrication, yields implant restorations with accuracy comparable to that of conventional impressions [17,18]. Together, these findings support growing evidence that fully digital approaches can achieve prosthetic precision comparable to that of established conventional techniques [17,19].

In contrast to prosthetic accuracy, clear advantages in clinical efficiency were observed in the digital test group. Chairside time for both impression taking and prosthesis placement was significantly reduced compared with the conventional CSCRP workflow. Intraoral digital scanning required less time than conventional PVS impressions, a finding consistent with previous reports demonstrating reduced procedure time and improved patient comfort with digital impressions [18,20]. Our results suggest that adopting a digital workflow can substantially enhance clinical efficiency, with potential benefits for both patient comfort and overall treatment productivity.

Prosthesis placement within the CL-SRP protocol was simplified, as the absence of a cementation step and associated cleanup allowed the definitive crown to be secured with minimal additional procedures. This streamlined sequence may also reduce the risk of cement-related complications or technical errors, thereby facilitating a safer and more predictable procedure [10,11]. In contrast, the CSCRP approach required intraoral cementation of the crown onto the abutment, followed by removal of the abutment–crown assembly for thorough extraoral removal of excess cement. The assembly then had to be reseated and secured with final screw tightening. These extra steps inevitably prolonged chairside time and introduced additional risks of biological or technical complications.

Within this context, the present findings indicate that the CL-SRP protocol can maintain prosthetic accuracy comparable to the conventional approach while simultaneously improving efficiency by streamlining the clinical sequence. This combined advantage of accuracy and reduced chairside time may enhance the patient experience and optimize workflow for clinicians.

The influence of each workflow on peri-implant soft tissues was assessed through analysis of gingival contour changes. No significant difference was observed between the control and test groups, indicating that both approaches were similarly effective in maintaining peri-implant mucosal health over the short follow-up period. Interestingly, the CL-SRP group showed less variability in gingival dimensional changes, which may reflect a more consistent soft tissue response. A plausible explanation is the “one-abutment–one-time” protocol, in which the definitive abutment is positioned once and left undisturbed, thereby preserving the integrity of the peri-implant mucosal seal. In contrast, repeated removal and reconnection of abutments in the conventional workflow, whether for impression taking or crown cementation, may have affected gingival rebound and contributed to greater variability [20,21].Although mean changes were comparable in both groups, the present study suggests that the CL-SRP system may promote more uniform gingival adaptation.

In this study, both the conventional CSCRP and digital CL-SRP workflows demonstrated MBL values that were well within the thresholds generally accepted as compatible with implant success [22,23]. Nonetheless, the test group exhibited significantly lower MBL during the prosthetic phase than the control group. This finding may be explained by the one-time abutment placement in the CL-SRP protocol, which minimizes disturbance at the implant–abutment interface and may thereby reduce crestal bone remodeling [24,25]. In contrast, the repeated removal and reconnection of abutments required in the CSCRP workflow could have contributed to localized micro-movements and subsequent bone adaptation. Although both groups remained within thresholds of early implant success, the observed mean difference of 0.24 mm in MBL may have clinical relevance over time, highlighting the importance of long-term, large-scale studies to assess its prognostic implications.

Another factor that may account for the present findings is the absence of cement use in the CL-SRP system. Residual cement—even when applied carefully—has been implicated in peri-implant tissue irritation and is associated with increased bone loss [26]. By eliminating the need for cement, the CL-SRP workflow avoids this well-documented biological risk.

Although the reduced bone remodeling noted in the test group is encouraging, the magnitude of change in both groups was small and remained within clinically acceptable limits. These short-term outcomes should therefore be interpreted with caution, as they do not establish long-term superiority. Continued follow-up and well-designed longitudinal studies will be needed to determine whether differences in MBL are sustained over time and whether they ultimately influence implant survival.

The present trial illustrates the ongoing shift in implant prosthodontics toward fully digital and cementless workflows. Within this context, the CL-SRP protocol achieved accuracy comparable to that of the conventional CSCRP approach. These findings contribute to the growing body of evidence that modern intraoral scanners, when combined with CAD/CAM fabrication, can deliver implant restorations with precision meeting established clinical standards. This aligns with prior studies that have dispelled concerns about the reliability of digital impressions [27,28,29] and supports evidence that patients prefer digital impressions for greater comfort and convenience [29,30,31].

Beyond accuracy, the CL-SRP approach integrates a cementless connection with a one-time abutment protocol, thereby minimizing the risks of excess cement retention and repeated abutment disconnections, both of which are associated with peri-implant complications [10,11]. The present results are consistent with recent reports indicating that cementless systems can achieve clinical outcomes comparable to, and in some cases surpassing, those of conventional cement-retained restorations. This convergence of findings across studies supports the practical adoption and feasibility of cementless protocols in implant dentistry.

Overall, the findings of this study suggest potential advantages for clinical efficacy. However, these results should be interpreted with caution and not regarded as definitive evidence of biological or prosthetic superiority.

Several limitations of the present trial should be acknowledged. First, the sample size was relatively small, including 35 patients and 40 single-implant restorations, which inevitably limits the generalizability of the findings. Although sufficient to detect significant differences in procedural time and marginal bone loss, the sample size was based on in vitro pilot data and may not fully reflect in vivo variability. Larger-scale, multicenter trials are needed to provide more robust and generalizable evidence for outcomes such as prosthetic adjustment and peri-implant soft tissue changes [30].

Second, the follow-up period was limited to approximately 6 months after implant placement. While this duration was adequate for assessing early clinical outcomes, it was insufficient for evaluating long-term complications, prosthetic maintenance, marginal bone stability, or implant survival. As a preliminary study, extended follow-up and larger-scale investigations with long-term observation are required to confirm the sustainability and clinical benefits of the digital CL-SRP workflow.

Third, this study compared the CL-SRP protocol with a CSCRP protocol, in which the crown is cemented to the abutment intraorally, then unscrewed and removed extraorally for thorough cement removal before being reattached and torqued to the implant fixture. Thus, although the final restoration was screw-retained, the CSCRP workflow was not entirely cementless. Therefore, the results should be interpreted as a comparison between two variants of screw-retained restorations—one cementless and one involving intraoral cementation with subsequent extraoral cement removal—rather than as a strict comparison between cemented and screw-retained prostheses. Future studies comparing screw-retained prostheses (without cement) using a digital workflow with those using CL-SRP are warranted.

Fourth, the study design combined two variables in the test group: a fully digital workflow and a CL-SRP system. This makes it difficult to isolate the contribution of each factor to the observed outcomes. The CSCRP protocol was used as the control group, as it reflects the most common clinical workflow, thereby allowing for a direct comparison with current standard practice. The primary objective of this preliminary study was to evaluate the combined impact of digitalization and cementless protocols. CSCRP was selected as the control group to reflect the mixed characteristics of screw–cement-retained fixation widely used in the Korean clinical setting. Further studies with separate comparisons are required to clarify the effects of each component.

Additionally, the lack of patient-reported outcomes, such as satisfaction or comfort, limits assessment of the clinical benefits of the compared workflows from the patient’s perspective. Finally, the findings of this study were restricted to single-implant restorations in posterior sites with favorable angulation. These results may not be generalizable to more complex clinical cases, such as full-arch rehabilitations or implants placed at challenging angulations. Further research is required to verify the efficacy and feasibility of both systems in complex clinical scenarios.

## 5. Conclusions

This randomized controlled clinical trial demonstrated that the fully digital CL-SRP workflow produced prosthetic outcomes comparable to the conventional CSCRP approach in single posterior implant restorations. The digital CL-SRP workflow significantly reduced impression and placement times while maintaining prosthetic accuracy and fit. Furthermore, the elimination of cementation and the implementation of a one-abutment–one-time protocol contributed to reduced chair time and favorable early peri-implant bone preservation. Collectively, these preliminary findings highlight the clinical feasibility of the CL-SRP workflow in enhancing procedural efficiency and peri-implant health. Future multicenter trials with larger sample sizes and extended follow-up are required to validate its durability and establish broader clinical applicability.

## Figures and Tables

**Table 1:** Time assessment for clinical procedure time.

Procedural Stage	Control Group	Test Group
Impression-taking (IT) time	- Removal of healing abutment - Placement of impression coping - Impression-taking with conventional material - Reattachment of healing abutment	- Removal of scan healing abutment - Base abutment torque check - Replacement with scan body - Scanning of the prepared implant site
Prosthesis placement (PP) time	- Removal of healing abutment - Initial abutment–crown trial fitting - Cementation - Retrieval of abutment–crown for excess cement removal - Final screw tightening - Resin sealing of the screw access hole	- Removal of the healing cap - Initial prosthesis trial fitting - Screw tightening to final torque - Resin sealing of the screw access hole

**Table 2:** Baseline characteristics.

Variable	Control Group (Implant N = 20)	Test Group (Implant N = 20)	Total (N = 40)	*p*-Value
Age (Mean ± SD)	60.6 ± 13.5	63.3 ± 11.5	61.9 ± 12.5	0.531
Gender (M:F)	9:9	12:5	21:14	0.369
Site				
Mandible	12 (60.0%)	12 (60.0%)	24 (60.0%)	1.000
Maxilla	8 (40.0%)	8 (40.0%)	16 (40.0%)	1.000
Molar	14 (70.0%)	13 (65.0%)	27 (67.5%)	1.000
Premolar	6 (30.0%)	7 (35.0%)	13 (32.5%)	1.000

All variables were compared using the chi-square test, except for age, which was assessed using an independent samples *t*-test. SD: Standard deviation; *p*-value < 0.05 considered statistically significant. M: Male; F: Female.

**Table 3:** Clinical outcomes.

	Control (N = 20)	Test (N = 19)	Mean Difference (95% CI)	*p*-Value	Cohen’s d
Impression taking (IT) time (s)	466.10 (96.11)	261.58 (119.11)	204.52 (147 to 272)	<0.001 *	1.890
Prosthesis placement (PP) time (s)	918.25 (128.46)	259.21 (95.73)	659.04 (582 to 761)	<0.001 ^†,^*	5.818
Prosthesis adjustment (PA) time (s)	444.90 (352.37)	329.58 (228.34)	115.32 (-52 to 239)	0.211	0.388
Total prosthetic time (s)	1363.15 (347.11)	588.79 (270.32)	774.36 (605 to 932)	<0.001 *	2.489
Adjustment volume (mm^3^)	3.38 (2.46)	1.96 (1.27)	1.42 (−0.793 to 2.199)	0.474	0.725
Gingival shape change (μm)	219.33 (187.79)	153.45 (50.16)	65.88 (−9.447 to 16.176)	0.966	0.479
Marginal bone loss (mm)	0.71 (0.33)	0.47 (0.28)	0.24 (0.035 to 0.437)	<0.05 ^†,^*	0.784

Data are presented as Mean (Standard deviation), and *p*-values were calculated using independent *t*-tests or Mann–Whitney U tests, depending on normality. † is used to indicate that an independent *t*-test. * is used to indicate that *p* < 0.05, signifying statistical significance.

## Data Availability

The de-identified participant data, statistical code, and related materials are available upon reasonable request from the corresponding author.
